# Perspectives on the current diagnostic and treatment paradigms in secondary hemophagocytic lymphohistiocytosis (HLH)

**DOI:** 10.1186/s13023-025-03698-0

**Published:** 2025-04-26

**Authors:** Leonard Naymagon, Philip Roehrs, Michelle Hermiston, James Connelly, Jeffrey Bednarski, Jaap-Jan Boelens, Shanmuganathan Chandrakasan, Blachy Dávila Saldaña, Michael M. Henry, Prakash Satwani, Anish Ray, Kelly Walkovich, David Teachey, Edward M. Behrens, Scott W. Canna, Ashish Kumar

**Affiliations:** 1 Mount Sinai School of Medicine, Tisch Cancer Institute, 1470 Madison Avenue, New York, NY 10029 USA; 2 Stem Cell Transplant and Cellular Therapies, Division of Hematology and Oncology, Department of Pediatrics, University of Virginia, Charlottesville, VA USA; 3 Department of Pediatrics, UCSF Benioff Children’s Hospital and the Helen Diller Family Comprehensive Cancer Center, University of California, San Francisco, San Francisco, CA USA; 4 Division of Hematology and Oncology, Department of Pediatrics, Vanderbilt University Medical Center, Nashville, TN USA; 5 Division of Hematology and Oncology, Department of Pediatrics, Washington University School of Medicine, St. Louis, MO USA; 6 Department of Pediatrics, Transplantation and Cellular Therapies, Memorial Sloan Kettering Cancer Center, New York, NY USA; 7 Aflac Cancer and Blood Disorders Center, Department of Pediatrics, Children’s Healthcare of Atlanta, Emory University School of Medicine, Atlanta, GA USA; 8 Department of Pediatrics, George Washington University, Washington, DC USA; 9 Division of Blood and Marrow Transplantation, Children’s National Hospital, Washington, DC USA; 10 Center for Cancer and Blood Disorders, Phoenix Children’s Hospital, Phoenix, AZ USA; 11 Division of Pediatric Hematology, Oncology and Stem Cell Transplantation, Department of Pediatrics, Columbia University, New York, NY USA; 12 Cook Children’s Medical Center, Fort Worth, TX USA; 13 Division of Hematology and Oncology, Department of Pediatrics, University of Michigan, Ann Arbor, MI USA; 14 Division of Oncology, The Children’s Hospital of Philadelphia, Philadelphia, PA USA; 15 Division of Rheumatology, Children’s Hospital of Philadelphia, Perelman School of Medicine at The University of Pennsylvania, Philadelphia, PA USA; 16 Cancer and Blood Diseases Institute, Cincinnati Children’s Hospital Medical Center, University of Cincinnati College of Medicine, Cincinnati, OH USA

## Abstract

Improved awareness of hemophagocytic lymphohistiocytosis (HLH) among clinicians has led to an increase in its diagnosis. Often diagnosis is made based on the HLH- 2004 criteria. While these criteria have considerable strengths, they lack specificity and may be fulfilled in the setting of many pro-inflammatory disorders. Genetic defects affecting cellular cytotoxicity cause familial (primary) HLH. On the other hand, secondary HLH is more a pathophysiologic process common to many conditions, rather than a singular disease entity. Improved genetic, immunologic, and functional testing have changed not only the way we diagnose HLH, but also how we treat it. In 2004, there were few active agents and regimens. In 2024, there are multiple safe and effective targeted therapies. We have begun to understand that routine and immediate use of etoposide-based therapy in secondary HLH is likely not appropriate, and emerging cytokine-directed therapies may be more rational interventions. Moreover, it is recognized that identifying and treating the driver of secondary HLH is at least as important as treating the cytokine storm and immune dysregulation. Unfortunately, over-reliance on, and narrow interpretation of, the HLH- 2004 criteria can lead to overdiagnosis, misdiagnosis, and unneeded exposure to drugs that can be harmful. It is important that clinicians understand the limitations of the current diagnostic paradigms for secondary HLH, and the shortcomings of reflexive use of etoposide-based therapy. Herein we will discuss the pros and cons of the current paradigm for the recognition, diagnosis, and treatment of secondary HLH.

## Background and introduction

Hemophagocytic lymphohistiocytosis (HLH) refers to a syndrome of excessive and maladaptive immune activation [1]. Such a syndrome may arise in the setting of recurrent heritable mutations leading to immune dysfunction; a distinct genetic-pathophysiologic entity termed “familial” or “primary” HLH” [2]. More often, and with increasing frequency, the syndrome is diagnosed in the absence of intrinsic genetic defects; in cases of seemingly disproportionate and deleterious immune responses to provoking proinflammatory triggers. Such instances of systemic inflammation, which may be sparked by a wide array of stimuli (including but not limited to infection, malignancy, metabolic, and rheumatologic disease), are often labeled “secondary HLH”, and now represent the majority of diagnosed HLH cases [3]. Whereas a diagnosis of primary HLH requires the clinical features of immune activation and, critically, the identification of a pathologic genetic mutation, secondary HLH is not a discrete entity and lacks any such definitive diagnostic finding.

Currently, the diagnosis of HLH is often based upon a set of guidelines known as the HLH- 2004 criteria [4, 5]. These guidelines were initially developed as inclusion criteria for a clinical trial investigating therapy for largely primary HLH in children. The HLH- 2004 criteria have certainly had strengths and benefits, including raising awareness of this previously little-known entity, offering unfamiliar clinicians a readily available and more straightforward means of approaching a complex condition, affording a consistent benchmark to allow comparisons across trials, and forming a basis from which to refine future understanding. However, there are limitations to the specificity and thus the applicability of these criteria.

Over time, the HLH- 2004 criteria have become the de facto clinical definition of HLH. This belies the fact that the HLH- 2004 criteria are not a set of specific, rigorously tested, or naturally distinct features of HLH, and were not meant to be disease-defining. Indeed, none of the components of these criteria are specific to HLH, and the criteria may be routinely fulfilled by many other entities which share the inflammatory features of HLH [6]. This may be leading to an increasing over-implication of HLH, resulting in missed or muddled diagnoses of relatively more common inflammatory conditions (such as hematologic malignancies, infections, and rheumatologic conditions). Notably, the original description of the HLH- 2004 criteria specifically caution against applying them in situations of malignancy or infection.

Furthermore, because of their association with the HLH- 2004 clinical trial, fulfillment of these criteria is often cited as sufficient rationale for the use of dexamethasone and etoposide in a wide array of clinical situations which happen to “check the boxes” prescribed in the originating study protocol [5]. This reflexive application of the HLH- 2004 criteria to trigger therapy is concerning, as the majority of patients in the trial had “primary/familial/genetic HLH” and were universally pediatric, whereas the majority of presumed HLH diagnoses made today are not genetic in origin and are often made among adult patients. There are no comparable prospective data, and scant if any retrospective data, to suggest that etoposide is effective and should play a front-line role in treating adults or children with non-genetic/secondary HLH [7]. There is concern that the HLH- 2004 criteria are being used as blanket justification for the use of etoposide-based therapies in instances where such therapies do not have a well-established place, leading to unnecessary and likely harmful treatments [8].

Herein, we will reassess the current paradigm for the recognition, diagnosis, and treatment of HLH, particularly secondary HLH. Among patients with primary HLH, the presence of an identifiable immune-dysregulating gene mutation acts as a gold-standard for diagnosis. The absence of such a clear litmus test in secondary HLH makes any diagnostic paradigm problematic. Over-reliance on a single non-specific paradigm may lead to inaccurate or incomplete diagnostic conclusions. Based on these inadequate diagnostic approaches, patients may be deemed candidates for a largely unfounded treatment. Our goal is not to discredit the current diagnostic paradigms, but rather to help clarify the contexts in which they may be applied. Specifically, we emphasize that secondary HLH is a description of an inflammatory state arising in the setting of another condition, rather than a specific disease entity (much as shock is the description of a physiologic state which may be the result of several diverse pathologic processes). Above all, we hope to motivate clinicians to think critically whenever a diagnosis of HLH is suggested (regardless of the age of the patient), and avoid routinely committing to a “primary HLH paradigm” in cases of secondary HLH.

## The HLH- 2004 criteria–a problem of specificity

In recent years the HLH- 2004 criteria have transformed from inclusion criteria for a clinical trial, into a disease-defining dogma, regarded by many clinicians as being nearly pathognomonic of familial (primary) HLH [9]. Certainly, a diagnostic framework is needed to help guide when HLH should be considered in differential diagnoses. Such an algorithm is particularly essential in situations without prior family history or knowledge of mutations in the given patient, as well as to identify potential cases meriting genetic testing (or necessitating urgent intervention while awaiting the results of genetic testing). While the HLH- 2004 criteria are useful in helping clinicians consider HLH in their differential diagnoses, and perhaps in helping recognize instances of maladaptive inflammation, their utility is akin to that of a screening test, not a confirmatory diagnostic test. These criteria are sensitive, however they are so sensitive as to be fulfilled by nearly any sufficiently pro-inflammatory process [6].

This lack of specificity is manifest when examining each individual criterion in earnest (Tables 1 and  2). Fever is a common manifestation of immune/inflammatory responses of all etiologies [10]. Similarly, cytopenias may be a consequence of any profoundly inflammatory state. Among patients in intensive care units (ICUs), 50–70% will have hemoglobin < 9 g/dL, up to 60% will have thrombocytopenia, and 7% or more will have leukopenia [11]. Hypofibrinogenemia may result from liver injury or disseminated intravascular coagulation (DIC), both common potential consequences of any/all fulminant inflammatory states, and may occur in over a third of patients admitted to ICUs for any indication [12]. Triglycerides are non-specific acute phase reactants and the triglyceride cutoff used in the HLH- 2004 criteria is low enough to be within range of baseline levels among a significant proportion of adult patients [13, 14]. Splenomegaly is a frequent finding in many of the conditions which share a differential diagnosis with HLH including hematologic malignancy, certain infections (viral, fungal, protozoal), and a number of rheumatologic disorders [15].

**Table 1:** The HLH- 2004 criteria for diagnosis of HLH are depicted

Inclusion criteria for the HLH- 2004 clinical trial
A molecular diagnosis in HLH-causative genes OR at least one affected sibling OR fulfillment of at least 5/8 of below diagnostic criteria:
Fever ≥ 38.5 C
Cytopenias affecting at least 2 of 3 lineages—Hemoglobin < 9 g/dL—Platelets < 100,000 per mL—Neutrophils < 1,000 per mL
Hypertriglyceridemia (fasting: > 265 mg/dL) or hypofibrinogenemia (< 150 mg/Dl)
Splenomegaly
Ferritin > 500 ng/ml
sIL- 2r (sCD25) > 2400 u/ml
Low or absent NK-cell activity
Hemophagocytosis in bone marrow or other tissue

Abbreviations: HLH–hemophagocytic lymphohistiocytosis; NK-cell–natural killer cell; sIL- 2r–soluble interleukin- 2 receptor [1]

**Table 2:** Limitations of the HLH- 2004 Criteria

Limitations of the HLH- 2004 criteria	
Criterion	Limitation(s)
Fever	A common manifestation of immune/inflammatory responses of all etiologies. [10]
Cytopenias	May be a consequence of any profoundly inflammatory state. Among patients hospitalized in ICUs, 50–70% will have anemia to < 9 g/dL, up to 60% thrombocytopenia, and 7% or more will leukopenia. [11]
Hypofibrinogenemia	May result from liver injury or DIC, both common consequences of any/all fulminant inflammatory states, and may occur in over a third of patients admitted to ICUs for any indication. [12]
Hypertriglyceridemia	Non-specific acute phase reactants (not unlike ESR or CRP). The cutoff used in the HLH- 2004 criteria is low enough to be within range of baseline levels among a significant proportion of adults in developed countries. [13, 14]
Splenomegaly	A frequent finding in many of the entities which share a differential diagnosis with HLH including hematologic malignancy, viral infection, and a number of rheumatologic disorders. [15]
Hyperferritinemia	The HLH- 2004 ferritin cutoff demonstrated a specificity of merely 0.3% for HLH (in a critical care population wherein pretest probability may be higher than in lower acuity settings) [16]. Among a cohort of 1055 adult patients with serum ferritin > 5000 ng/mL the prevalence of diagnosed HLH was 6.5%, with prevalence only reaching 50% as serum ferritin approached 90,000 ng/mL [17]. In these cohorts and others, a wide variety of common non-HLH conditions have been associated with profound hyperferritinemia including sepsis, hematologic malignancy, rheumatologic disease, liver injury, and kidney failure
Soluble IL- 2 receptor	May be increased in any process involving T-cell activation (including sepsis, hematologic malignancy, rheumatologic disease, sarcoidosis, and inflammatory bowel disease) [20–24]. Among 132 patients with soluble IL- 2 receptor levels checked for evaluation of HLH, the specificity of the HLH- 2004 cutoff value was 38.8%, with an AUC for the corresponding ROC of 0.69, and no significant difference in levels when comparing patients with HLH, and non-HLH patients with sepsis, hematologic malignancy, or rheumatologic disease [25]
NK-Cell Activity	A cohort of 34 secondary HLH patients demonstrated an “activated NK phenotype profile” similar to inflammatory conditions such as sepsis or rheumatologic disease [27]. Among a cohort of 311 HLH patients, those with primary disease had significantly lower NK cell activity than those with secondary disease, with many secondary HLH patients exhibiting NK activities within the normal range [28]. In primary HLH, the NK-cell cytotoxicity assay has displayed low reliability with a poor AUC of 0.69 at the diagnostic ROC [29]
Bone Marrow Hemophagocytosis	A non-specific finding which may be encountered in a wide array of critically ill patients, including as many as 65% of autopsied ICU deaths, and 44% of autopsied inpatients [30, 31]

Abbreviations: AUC – area under curve; DIC – disseminated intravascular coagulation; CRP – C-reactive protein, ESR – erythrocyte sedimendation rate; HLH – hemophagocytic lymphohistiocytosis; ICU – intensive care unit; ROC – receiver operator curve

Hyperferritinemia often draws the attention of clinicians, and profound elevations of serum ferritin in a critically ill patient is often the initial impetus for many HLH evaluations. However, the HLH- 2004 ferritin cutoff has been reported to have a specificity of merely 0.3% for HLH even in critical care settings [16]. Among a cohort of 1055 adult patients with serum ferritin > 5000 ng/mL the prevalence of HLH was 6.5%, with prevalence only reaching 50% as serum ferritin approached 90,000 ng/mL [17]. It has been suggested that hyperferritinemia may be a more specific marker of HLH among pediatric patients, particularly if a higher cutoff is used. For instance, in a 330 patient pediatric cohort, although the HLH- 2004 ferritin cutoff had very poor specificity, a cutoff of > 10,000 ng/mL demonstrated 96% specificity (although of note this cohort contained only 10 patients diagnosed with HLH) [18]. However, among a cohort of 163 hospitalized children exceeding the HLH- 2004 cutoff ferritin level, only 8 (4.9%) were diagnosed with HLH, and even at a cutoff value of > 10,000 ng/mL the positive predictive value for ferritin remained only 18% [19]. In these cohorts and others, a wide variety of common non-HLH conditions have been associated with profound hyperferritinemia including sepsis, hematologic malignancy, rheumatologic disease, liver injury, and kidney failure.

HLH- 2004 helped introduce two new markers that have since become well-known features of the diagnostic paradigm; soluble IL- 2 receptor (soluble CD25), and NK-cell activity [4]. Among many clinicians, these tests carry the expectation of being more specific than those mentioned above. However, this expectation does not appear to be well founded. Soluble IL- 2 receptor may be increased in any process involving T-cell activation (including but not limited to sepsis, hematologic malignancy, rheumatologic disease, sarcoidosis, and inflammatory bowel disease) [20–24]. In a cohort of 132 consecutive patients with soluble IL- 2 receptor levels checked for evaluation of HLH, the specificity of the HLH- 2004 cutoff value was 38.8%, with an AUC for the corresponding ROC of 0.69 [25]. Additionally, no significant difference in levels were identified when comparing patients with HLH, to those non-HLH patients who carried diagnoses of sepsis, hematologic malignancy, or rheumatologic disease [25]. It is possible that a higher soluble IL- 2 cutoff may be more specific for HLH, particularly among pediatric patients, although this remains unclear.

Defects in genes regulating cytotoxic lymphocyte function (leading to suppressed NK cell activity) are hallmarks of primary HLH [26]. However, it is less clear if the popular NK cell activity assay is a valid marker for the presence of these genetic defects, nor what (if any) value it holds in secondary HLH. Notably, relative deficiency of peripheral NK cells and intrinsic defects in NK cell activity affect this assay, and both can occur transiently in a great number of inflammatory states. In a cohort which included 34 secondary HLH patients, and 34 non-HLH controls, NK cell cytotoxicity did not differ significantly between groups, with the secondary HLH patients demonstrating an “activated NK phenotype profile” similar to inflammatory conditions such as sepsis or rheumatologic disease [27]. Among a cohort of 311 HLH patients, those with primary disease had significantly lower NK cell activity than those with secondary disease, with many secondary HLH patients exhibiting NK activities within or near the normal range [28]. Even in primary HLH, the NK-cell cytotoxicity assay has displayed low reliability with a poor AUC of 0.69 at the diagnostic ROC, demonstrating the non-specific nature of this test [29].

Finally, bone marrow hemophagocytosis, despite lending its eponym to HLH, is also a non-specific finding that may be encountered in a wide array of critically ill patients, including as many as 65% of autopsied ICU deaths, and 44% of autopsied inpatient deaths [30, 31].

Recognition of the shortcomings of the HLH- 2004 criteria has led to interest in developing other criteria, the HScore perhaps the most well-known among these [32]. Some data does suggest improved diagnostic accuracy with the HScore [33]. However, as 7 of the HScore’s 9 components are either the same as, or similar to, those of the HLH- 2004 criteria, it is prone to similar pitfalls. By offering a continuous risk score rather than a categorical diagnosis, the HScore may at least offer some greater nuance and the ability to quantify diagnostic probability to some degree.

In primary HLH, the presence of a gold-standard confirmatory test obviates over-reliance on clinical criteria. The absence of any such gold-standard diagnostic in secondary HLH, when combined with the non-specific nature of the syndrome, makes reliable diagnosis a formidable, and to-date inadequately addressed challenge. These notions call into question whether secondary HLH can truly be defined as a unique entity, or whether it is simply a label for excessive and maladaptive inflammation provoked by any external cause.

**Fig. 1:**
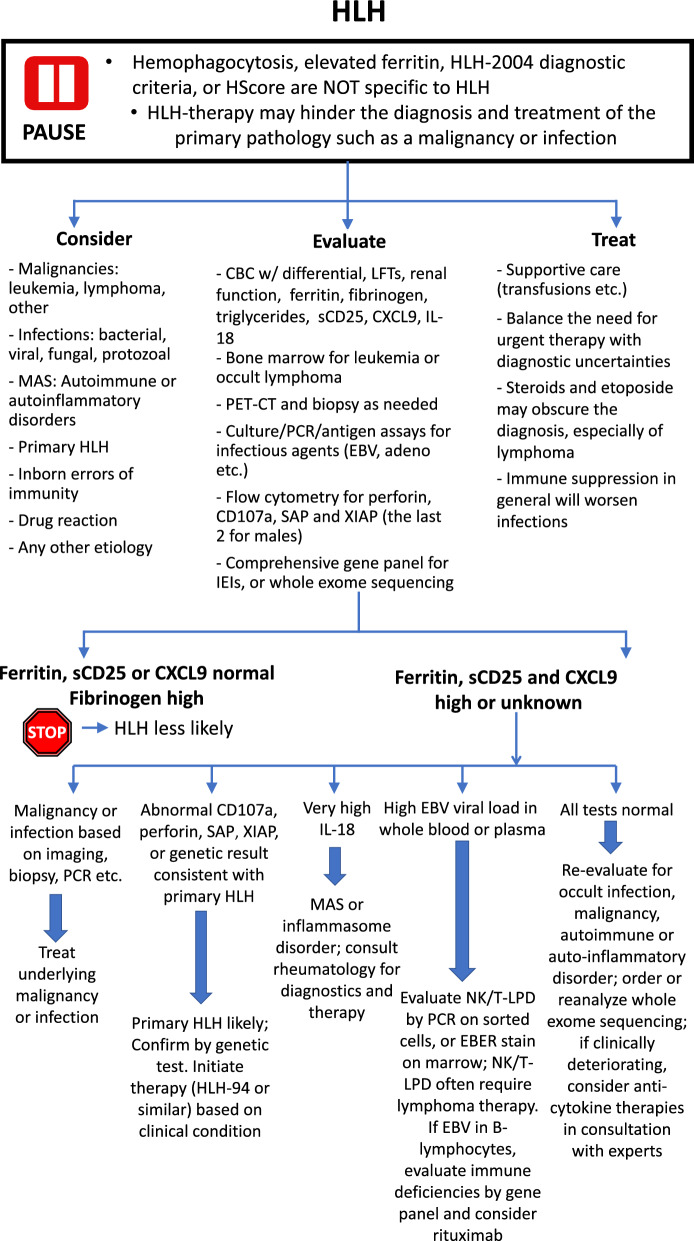
A proposed algorithm for evaluation and management of suspected HLH. It must be noted that there remains insufficient data to make definitive recommendations regarding diagnosis and management of HLH and the above algorithm is based on author consensus. Above all it must be emphasized that patients with hyper-inflammatory phenotypes of unclear etiology should undergo exhaustive workup for malignancy, infection, and rheumatologic disease, before etoposide-based therapy is considered. *Anti-inflammatory therapies may include agents such as steroids and/or cytokine-directed agents (such as anakinra, tocilizumab, emapalumab, or ruxolitinib). Adapted from the HLH Center of Excellence at Cincinnati Children’s Hospital Medical Center (original algorithm can be downloaded here https://www.cincinnatichildrens.org/service/h/hlh/clinical/test)

*Summary Statement*: The HLH- 2004 criteria are not specific for primary HLH without confirmatory genetic testing and may be fulfilled by many pro-inflammatory conditions. As such, patients without identifiable genetic mutations in HLH-causative genes should continue to be investigated for alternative diagnoses. In these latter situations (secondary HLH) it is critical to exhaustively search and identify the driver leading to the pro-inflammatory state (Fig. 1). Secondary HLH must be recognized as a pathophysiologic process that may accompany several inflammatory disorders, rather than a discrete, independent diagnosis.

## Secondary HLH–overuse of etoposide-based therapy

The HLH- 94 and HLH- 2004 clinical trials established dexamethasone and etoposide as effective first line therapy for primary HLH [5, 34]. Since the inclusion criteria for these trials have come to define HLH, fulfillment of these criteria has been widely accepted as an indication for treatment with dexamethasone and etoposide. This may be problematic given both the very non-specific nature of these criteria and given the limited generalizability of the HLH- 94 and HLH- 2004 clinical trials. The cohorts in these trials included substantial proportions of patients with confirmed primary HLH, and although many patients with secondary disease were included, this case-mix may no longer be reflective of the changing landscape of HLH diagnosis (wherein increased awareness of the condition has led to its increased implication as a secondary phenomenon, and a preponderance of secondary diagnoses). More notably, these were exclusively pediatric trials, and the generalization of their findings to adult patients has not been well founded. The Histiocyte Society has cautioned against over-use of etoposide, particularly in cases outside the narrow inclusion criteria of the HLH- 94 and HLH- 2004 clinical trials [35].

There remains a lack of data regarding the role of etoposide-based therapy in secondary HLH, particularly in adults, in whom HLH is almost universally secondary. Indeed, there is a virtual absence of any prospective data investigating the use of dexamethasone and etoposide in adults with HLH, and even rigorous retrospective data are limited. This is becoming increasingly problematic the limited data on adult HLH which is emerging, demonstrate that it behaves as a fundamentally different phenomenon, with a seemingly more aggressive course, and with poorer survival outcomes regardless of therapy. In a single center retrospective study comparing children and adults with HLH, 70% (7/10) of pediatric patients survived one year, compared with only 33% (7/21) of adult patients [36]. These findings are similar to reported survival rates in the broader pediatric and adult literature. Whereas 62% of the pediatric cohort in HLH- 2004 remained alive at 5 years post-enrollment, most adult cohorts have reported far more dismal outcomes. Adult HLH patients have demonstrated striking early mortality with 20–40% of adult patients dying within 30 days of diagnosis [37–39]. Median survival times among adult patients have been reported to be in the range of 1–4 months, with fewer than a third of patients surviving follow-up in most studies [7, 39–42]. This difference in outcomes may be due at least in part to differing etiologies and pathologies of HLH among pediatric and adult patients; with primary HLH and rheumatologic etiologies accounting for a relatively larger proportion of pediatric cases, and malignant and infectious etiologies accounting for a relatively larger proportion of adult cases.

There is little evidence that etoposide-based therapy is of benefit in adults with secondary HLH. We were able to identify only 5 small retrospective studies which included mention of a comparison between patients who received etoposide-based therapy, and those who did not (most often patients treated only for the underlying cause of secondary HLH), (Table 3). In a retrospective cohort of 62 adults with secondary HLH assessed for relevant prognostic factors, the overall survival (OS) among those who received etoposide-based therapy (n = 21) was similar to those who did not (n = 41) [40]. In a comparable cohort of 68 adult HLH patients OS was not significantly different between the etoposide (n = 32) and no-etoposide (n = 36) groups [41]. In a study investigating predictors of early death (within 30 days of diagnosis) among 162 adult patients with secondary HLH, the 81 patients who received etoposide-based therapy did not demonstrate significantly improved 30-day survival relative to the 81 who did not [38]. Although the use of etoposide was not associated with better survival in univariate analysis (*p* = 0.079), in multivariate analysis, the absence of etoposide use was associated with a worse prognosis (*p* = 0.04). A study investigating prognostic factors and outcomes among a 64 patient adult HLH cohort (16 of whom received etoposide based therapy) found that etoposide did not have an impact on OS [39]. In a retrospective study of 90 adult secondary HLH patients comparing outcomes among those who received etoposide-based therapy (n = 42) to those who did not (n = 48), use of etoposide was not associated with an improvement in survival [7]. Notably, all above studies were observational and none were controlled. Additionally, outcomes may have been skewed by use of etoposide in the most ill or refractory patients. The most common causes of HLH across these studies were malignancy, infections, and autoimmune disease (with confirmed primary instances of HLH exceedingly rare). The studies were not sufficiently powered to draw conclusions regarding the efficacy of etoposide based on etiology of HLH, although patients with underlying malignancy did consistently demonstrate the poorest outcomes (whether or not etoposide was given).

**Table 3:** Studies describing the use of etoposide-based therapy in secondary HLH. We were able to identify only 5 small retrospective studies which included any mention of a comparison between patients who received etoposide-based therapy, and those who did not. Those, as well as one additional study describing use of the HLH- 2004 protocol in adult secondary HLH (Bubik et al.) are summarized above. Note all above studies were uncontrolled and may have been skewed by use of etoposide in the most ill or refractory patients

Studies describing the use of etoposide-based therapy in secondary HLH	
Study (year)	Notable findings
Parikh et al. [40]	In a retrospective cohort of 62 adults with secondary HLH assessed for relevant prognostic factors, the OS among those who received etoposide-based therapy (n = 21) was similar to those who did not (n = 41) [40]. The underlying cause of HLH was malignancy in 32 patients (52%), infection in 21 (34%), autoimmune disease in 5 (8%), and idiopathic in 4 (6%)
Arca et al. [38]	In a study investigating predictors of early death (within 30 days of diagnosis) among 162 adult patients with secondary HLH, the 81 patients who received etoposide-based therapy did not demonstrate significantly improved 30-day survival relative to the 81 who did not [38]. Although the use of etoposide was not associated with better survival in univariate analysis (p = 0.079), in multivariate analysis, the absence of etoposide use was associated with a worse prognosis (p = 0.04). Hematological malignancies (n = 75, 46%), infections (n = 40, 25%), and multicentric Castleman disease (n = 17, 10%) were the most common identified triggers
Schram et al. [41]	In a cohort of 68 adult HLH patients OS was not significantly different between the etoposide (n = 32) and no-etoposide (n = 36) groups [41]. Underlying disorders included malignancy in 33 patients (49%), infection in 22 (33%), autoimmune disease in 19 (28%) and idiopathic HLH in 15 (22%)
Apodaca et al. [39]	A study investigating prognostic factors and outcomes among a 64 patient adult HLH cohort (16 of whom received etoposide based therapy) found that etoposide did not have an impact on OS [39]. Causes of HLH included malignancy in 33 patients (52%), infection in 17 (27%), autoimmune in 3 (5%), familial in 1 (2%), idiopathic in 10 (16%)
Bubik et al. [42]	A retrospective study of 31 adult HLH patients treated according to the HLH- 2004 protocol demonstrated a median OS of 3.2 months, and 1-year overall survival of 35% [42]. HLH etiology included malignancy (*n* = 9, 29%), autoimmune (*n* = 8, 26%), infection (*n* = 8, 26%), and idiopathic (*n* = 6, 19%)
Naymagon et al. [7]	In a retrospective study of 90 adult secondary HLH patients comparing outcomes among those who received etoposide-based therapy (n = 42) to those who did not (n = 48), use of etoposide was not associated with an improvement in survival [7]. Causes of HLH included infection in 63 patients (70%), malignancy in 44 (49%), rheumatologic disease in 13 (14%). Thirty patients (33%) had multiple concurrent causes

Abbreviations: HLH – hemophagocytic lymphohistiocytosis; OS – overall survival

Among pediatric patients, the use of etoposide-based therapy is certainly better established, particularly in the setting of primary HLH [5, 34]. However, with the increased awareness of HLH, we are seeing more and more children prematurely diagnosed with HLH, and inappropriately treated with dexamethasone and etoposide with the default presumption of primary HLH. Such measures often lead to potentially harmful use of this therapy in settings where it may not be warranted [8]. In many instances, reflexive use of dexamethasone and/or etoposide may delay or compromise treatment for the root cause of the inflammation or may further obscure the underlying diagnosis [8]. Even among children, primary HLH is a far less common entity than hematologic malignancy, infection, or autoimmunity, and these conditions must be considered and thoroughly investigated [43, 44]. Sometimes treatment cannot wait for genetic confirmation of primary HLH, which can take several weeks. In these situations, in children, steroids and etoposide may be considered after biopsies to rule out malignancy have been collected and while awaiting confirmatory results for primary HLH (flow cytometry and genetics). Such an approach may be more questionable in adults where the probability of primary HLH is extremely low.

Nevertheless, many adult clinicians may pursue a limited course of etoposide in those cases where the hyperinflammatory state is not adequately controlled via other interventions (such as treating the underlying trigger of secondary HLH, and/or other anti-inflammatory therapies such as steroids or anti-cytokine agents). The benefit of such an approach is not well established however at least some reports of favorable responses do exist [45]. In all such cases the potential benefits and risks of etoposide should be carefully weighed and reasonable alternatives considered. In those instances where etoposide is used in the management of adult secondary HLH, it may not be ideal to pursue the pediatric dosing described in the HLH- 94 protocol, and dosage may need to be reduced or otherwise adjusted particularly in the setting of relevant comorbidities [9]. The need for continuous real-time evaluation of the patients´ response to therapy and need for escalation, de-escalation, or change of strategy, should be dynamic, particularly as the balance between hyperinflammation and an immunocompromise may change rapidly.

A specific instance where steroids and etoposide may have therapeutic value outside of primary HLH, is in EBV-associated lymphoproliferative disorders (LPDs), which include EBV-HLH. Although this group of diseases are often incorrectly labeled as infection-associated (secondary) HLH, the pathophysiology is more akin to EBV-induced lymphomas [46]. These EBV-associated T- and NK-cell LPDs occur more frequently in people of Asian ethnicities and among indigenous peoples of the Americas. The clinical features of these disorders are identical to those of HLH i.e. fulfilling the HLH- 2004 criteria, and as such the term EBV-HLH or HLH-secondary to EBV infection is often used. While the initial treatment regimens used for these conditions may include dexamethasone and etoposide, usually lymphoma-based therapies are required for disease control [47].

Some benefit may also be derived from the incorporation of etoposide into chemotherapy regimens for treatment of lymphoma associated HLH [48]. Etoposide is a component of multiple well established treatment regimens for management of aggressive lymphomas (most notably the CHOEP and EPOCH regimens). Thus, much of the benefit of etoposide in this setting may be from its anti-lymphoma effect rather than any specific anti-HLH. Dose adjusted EPOCH regimens have shown efficacy in lymphoma associated HLH [48]. DEP (doxorubicin-etoposide-methylprednisolone) based regimens have also shown efficacy in lymphoma and EBV associated HLH [49–51]. The role of such etoposide-based chemotherapy in cases of HLH not associated with lymphoma nor EBV is likely much more limited although has been reported [52]. Critically, the prognosis of secondary HLH varies significantly depending on the underlying cause, with malignancy associated cases carrying the poorest prognosis, and other cases carrying a relatively more favorable prognosis (with cases due to rheumatologic disease tending to have the most favorable outcomes). The majority of patients with malignancy associated HLH have aggressive lymphomas, and etoposide is a core component of chemotherapy regimens for many of these patients (albeit at dosages typically higher than in the HLH- 94 protocol) [48]. Etoposide remains a component of rational and effective therapy in this subgroup of HLH patients, though its particular use in this population may impose an unfavorable skew to its reported efficacy in many studies (as these tend to among the most ill HLH patients, with particularly poor pre-treatment prognosis).

*Summary Statement*: There are insufficient data to support the broad and routine use of dexamethasone and etoposide-based therapy in secondary HLH (especially adult secondary HLH). Fulfillment of the HLH- 2004 criteria alone is not sufficient to merit commitment to this combination therapy, particularly among adult patients, and among those in whom a clear and treatable disease appears to be the precipitating factor for HLH. Regardless of the patient’s age, reflexive or premature use of etoposide and dexamethasone may mask an alternative diagnosis or may compromise treatment for the underlying cause of disease.

## Overemphasis of the HLH- 2004 criteria–missing the forest for the trees

Increased awareness of HLH, and of the HLH- 2004 criteria, has led to an increase in diagnoses [53]. In many circumstances, the label of HLH gets applied even in the presence of specific diagnosed inflammatory diseases. Most concerning are scenarios wherein patients with hyper-inflammatory presentations without evident etiology are labeled with “HLH” as the conclusive diagnosis without first completing a sufficiently extensive workup for other more common causes of systemic inflammation. We have seen patients treated primarily with etoposide-based therapy, with subsequent diagnosis of underlying lymphoma or occult infection [8]. In such cases, premature invocation of HLH can lead to either suspension of diagnostic efforts to identify the underlying etiology of inflammation, or can further obfuscate the underlying etiology by partially treating or masking the true disease process [54]. The resultant delays in appropriate treatment may cause significant morbidity and mortality. In such instances secondary HLH should be regarded as a description of a maladaptive inflammatory state accompanying an underlying pathology, not a discrete diagnosis by itself (much as shock does not represent an ultimate diagnosis, but rather a physiologic consequence of many illnesses of sufficient severity). The focus in such cases should remain on thorough and extensive investigation for occult malignancy, infection, and autoimmune disease (Fig. 1). If there is a family history of HLH, or if genetic testing or flow cytometry–based immunologic assays are consistent with primary HLH, the diagnostic and treatment focus should shift toward HLH. In the absence of any such data, some other underlying pro-inflammatory entity should always be considered until relevant diagnostic efforts have been exhausted [8, 55].

Some hematologic malignancies are associated with markedly higher levels of inflammation than others and such maladaptive inflammation likely contributes to poor outcomes in many such cases. This clinical association has been termed “hematologic malignancy-associated HLH” (HM-HLH) and better diagnostic parameters are being developed to differentiate these hyper-inflammatory malignancies from those that simply fit the non-specific HLH- 2004 criteria [56]. One such tool, called the “optimized HLH-inflammatory index” (OHI) was shown to be superior to HLH- 2004 in identifying hematologic malignancies that were associated with higher-than-expected inflammation and worse prognosis. In such patients, the additional diagnosis of HM-HLH likely has utility in helping recognize maladaptive inflammation, and sparking consideration of anti-inflammatory therapies in conjunction with treatments directed at the underlying malignancy.

In contrast, in the majority of situations that simply fulfill the loosely defined HLH- 2004 criteria or HScore, the term HLH may sometimes add more confusion than clarity. Many patients who may have previously been regarded as having a principal diagnosis of severe infection or hematologic malignancy, are now frequently labeled as having severe infection “with HLH” or hematologic malignancy “and HLH”, should they happen to “check the boxes” of the HLH- 2004 criteria. The utility of affixing HLH to these diagnoses is in many ways unclear. A diagnosis of secondary HLH, particularly outside of the specific HM-HLH described above, often raises the question of which disease process should be treated first (HLH or the provoking entity), or if both should be treated simultaneously (and how potentially conflicting therapies may be reconciled). Delaying or compromising treatment for malignancy, infection, or another pro-inflammatory stimulus, to offer etoposide for HLH may be potentially catastrophic. For instance, adding immune suppression with steroids and/or etoposide to the antimicrobial regimen of a patient with “HLH” secondary to severe infection (such as disseminated adenovirus) may weaken the immune response and ultimately lead to a worse outcome.

In a patient with a known rheumatologic disorder, or if one is found during the course of the acute HLH illness, the term macrophage-activation-syndrome (MAS) may be more apt than HLH. Recent advances in understanding the pathophysiology of MAS have led to novel diagnostic tools (e.g. IL- 18) and therapeutic approaches aimed at targeting the cytokines involved [57–59]. Separately, immunotherapies such as chimeric antigen receptor (CAR) T-cell therapy are also associated with inflammatory states that share features included in the HLH- 2004 criteria. As novel immunotherapies are developed, newer forms of inflammatory phenomenon will likely be recognized, with many sharing elements of the non-specific HLH- 2004 criteria [60]. The terms cytokine-release-syndrome (CRS) and immune-effector-cell associated HLH-like syndrome (IEC-HS) are likely more appropriate in these situations, recognizing that there might be additional varieties of these iatrogenic inflammatory states, depending on the differences in the target or the therapy.

*Summary Statement*: Secondary HLH should be regarded as a description of a maladaptive inflammatory state accompanying other disorders, not as a discrete and independent diagnosis. The implication of secondary HLH (whether via the HLH- 2004 criteria or otherwise), should prompt efforts to identify the underlying cause of the hyper-inflammatory state, and treatments aimed at addressing that underlying cause. Even in children, contemplation of HLH should be accompanied by a thorough search for the more common causes of fulminant inflammation (such as hematologic malignancy, infection, or autoimmune disease). Premature or inappropriate use of etoposide-based therapy may obfuscate the underlying cause of inflammation (making accurate diagnosis more difficult), and/or cause significant morbidity by preventing, delaying, or compromising appropriate treatment for the underlying cause of inflammation.

## The potential of cytokine-directed therapy–a more rational approach

Although not diagnostic of any unique disease entity, the HLH- 2004 criteria have helped clinicians identify patients who may be suffering from inappropriate or excessive systemic inflammation. In this sense, rather than assigning the label of “secondary HLH” as a singular disease, this paradigm may simply help clinicians pick up on “HLH physiology”, a maladaptive proinflammatory state which may occur as the consequence of nearly any sufficiently inflammatory process. HLH physiology has also been referred to as a “cytokine-storm” [61, 62]. Indeed, it is a state of profound cytokinemia involving elevations in interferon-gamma (IFN-γ), tumor necrosis factor (TNF), interleukin- 1 (IL- 1), IL- 2, IL- 6, and IL- 18, among others [62]. The hypercytokinemia of HLH has become an attractive diagnostic and therapeutic target. It is possible that identification of a particular cytokine profile may be a more reliable way of diagnosing maladaptive inflammation or HLH physiology than the HLH- 2004 criteria or other similar paradigms [63]. This remains an ongoing area of investigation and development, with immediate clinical utility being hampered by the limited availability of relevant cytokine assays.

Nevertheless, the prospect of identifying pathologic cytokine signatures, and tailoring therapies to the specific cytokine derangements of individual patients, remains appealing. Cytokine-directed therapies such as anakinra, tocilizumab, ruxolitinib, and emapalumab have had demonstrated efficacy in HLH [64–67]. The IL- 1 antagonist anakinra has been shown to yield impressive responses and survival rates in patients with secondary HLH due to a wide array of underlying inflammatory triggers, in both pediatric and adult patients, including those with severe critical illness, and those who had previously been refractory to etoposide-based therapy [68–71]. The IL- 6 antagonist tocilizumab has been reported to yield similar responses in secondary HLH patients, and has drawn particular attention for its effectiveness in treating CRS following CAR-T therapy (a state that is physiologically similar, although not identical to HLH), and CRS in severe coronavirus disease 2019 (COVID- 19) [65, 72–74]. The JAK1/JAK2 inhibitor ruxolitinib has demonstrated efficacy among adult patients with secondary HLH, and among HLH patients relapsed or refractory to prior etoposide-based therapy [67, 75]. Additionally, there is some encouraging data supporting addition of ruxolitinib to standard chemotherapy protocols in the management of HM-HLH [76, 77]. The IFN-γ inhibitor emapalumab is effective in pediatric patients with primary HLH (including many who had previously failed etoposide-based therapy), and in patients with MAS [59, 78]. The key role of IFN-γ in HLH pathogenesis, and its activity relatively high up the cytokine cascade, makes it a particularly attractive target for HLH therapy, and this has been borne out in early reports of its real-world use (albeit largely in primary HLH) [79]. However, the high financial cost of the agent (even in comparison to other cytokine directed therapies) may hinder its use in resource limited settings [80].

Such therapies are particularly attractive because they are less immunosuppressive, less myelosuppressive, and less toxic than etoposide-based therapies and are therefore easier to reconcile with therapies targeting the underlying causes of secondary HLH. Their potential steroid sparing effect is also attractive and may reduce toxicity from long term steroid therapy. Importantly, these treatments do not directly impact the underlying pathology of malignancies such as lymphoma, thus preserving the ability to make accurate diagnoses. They also offer something beyond the “one-size-fits-all” approach which has pervaded previous thinking regarding HLH therapy and herald the possibility of rational targeted treatments which may be applied selectively to cases of pathologic hyperinflammation of a variety of causes. It is likely that such cytokine-directed therapies will replace etoposide as front-line interventions among patients deemed to have HLH (although etoposide will likely remain a frequent treatment in primary HLH) and may eventually have a broader role in the management of patients with severe hyperinflammation. Which patients are most likely to benefit from such therapies, which of these therapies should be given preferentially and on what basis (cytokine profiles, etiology of inflammation, etc.), and whether combinations may yield further benefit (and remain safe and tolerable) are yet to be determined.

*Summary Statement*: Anti-inflammatory therapies, via careful use of glucocorticoids and/or cytokine blockade, may be potential adjuncts to therapies directed at the underlying/provoking cause of secondary HLH. Such therapies are likely to be less toxic than etoposide-based therapies. It remains unclear however which patients are “sufficiently hyperinflammatory” to merit cytokine-directed therapies, how the optimal cytokine-directed therapy should be chosen, and whether such therapies may be combined.

## Conclusions

Primary HLH is a heritable genetic disorder resulting from defects in the cytotoxic immune response. Fulfillment of the HLH- 2004 diagnostic criteria should alert clinicians to consider primary HLH in the differential diagnosis in children, however, genetic evidence is still required to establish the diagnosis. In the absence of genetic mutations, the HLH- 2004 criteria describe a state of systemic inflammation, colloquially referred to as secondary HLH. Secondary HLH is often caused by an underlying proinflammatory pathology that must be identified and treated. While dexamethasone and etoposide are demonstrably effective in treating primary HLH, evidence supporting this combination therapy in secondary-HLH is lacking, and when misapplied this therapy may be harmful. Secondary HLH should be recognized more as a clinical manifestation of inflammation caused by some specific underlying primary diagnosis, instead of a conclusive diagnosis in itself. Treatments aimed at controlling the inflammatory cytokines associated with “HLH physiology” might be helpful in ameliorating excessive and maladaptive inflammation, however identification and definitive treatment of the underlying primary pathology is still required. A proposed algorithm for evaluation and management of suspected cases of HLH is shown in Fig. 1.

## Data Availability

Not applicable.
